# Demand‐Matched Self‐Efficacy and Perceived User Incivility in Healthcare: A Two‐Wave Study and an Exploratory Training Intervention

**DOI:** 10.1155/jonm/7488653

**Published:** 2026-10-07

**Authors:** Elena Cavallari, Ilaria Setti, Deirdre E. O’Shea, Valentina Sommovigo

**Affiliations:** ^1^ Department of Brain and Behavioural Sciences University of Pavia 27100 Pavia Italy unipv.eu; ^2^ Department of Work & Employment Studies University of Limerick V94 T9PX Limerick Ireland ul.ie; ^3^ Department of Economics and Management University of Pavia 27100 Pavia Italy unipv.eu

**Keywords:** bullet points, intervention, occupational well-being, self-efficacy, two-wave design, user incivility

## Abstract

**Aim(s):**

To examine which of three functionally distinct, demand‐relevant self‐efficacy beliefs showed the most consistent prospective association with baseline‐adjusted changes in perceived user (i.e., patients, relatives and visitors) incivility, psychosomatic symptoms and job satisfaction among healthcare workers, and whether a targeted training programme can strengthen this resource in acute nursing settings.

**Design:**

Two linked studies: Study 1 used a two‐wave change‐over‐time design, and Study 2 used an exploratory pre–post intervention design.

**Methods:**

In Study 1, 74 healthcare workers completed measures at two time points over three months. Standardised residualised change analyses examined whether three baseline self‐efficacy beliefs differing in their level of specificity and primary capability domain—self‐efficacy in managing challenging user behaviour, regulatory emotional self‐efficacy in managing negative emotions at work (RESE‐W) and trauma‐related coping self‐efficacy (CSE)—predicted changes in perceived user incivility, psychosomatic symptoms and job satisfaction. In Study 2, 22 nurses from acute units participated in a unit‐tailored training programme grounded in social cognitive theory. Pre–post analyses assessed changes in self‐efficacy for managing challenging user behaviour and related communication competencies.

**Results:**

In Study 1, higher baseline self‐efficacy in managing challenging user behaviour predicted more favourable changes in perceived user incivility, psychosomatic symptoms and job satisfaction. In contrast, CSE was associated only with psychosomatic symptoms, while RESE‐W showed no significant associations. In Study 2, self‐efficacy in managing challenging user behaviour showed a significant immediate pre–post increase following the intervention.

**Conclusion:**

Among the self‐efficacy beliefs examined, self‐efficacy in managing challenging user interactions showed the most consistent predictive pattern across the three outcomes. Preliminary findings indicate a short‐term increase in this psychological resource following brief, context‐sensitive training.

**Implications for Nursing Management:**

Assessing and supporting interaction‐focused self‐efficacy may complement broader organisational strategies for managing user incivility. Controlled studies are needed to determine whether targeted training produces durable improvements in occupational well‐being or care quality and whether it can be scaled across settings.

**Reporting Method:**

Study 1 adhered to the STROBE guidelines; Study 2 adhered to the TREND and TIDieR guidelines.

**Patient or Public Contribution:**

No patient or public involvement.

**Summary**
• The three efficacy beliefs showed differentiated prospective patterns. Self‐efficacy in managing challenging user behaviour was associated with baseline‐adjusted change across all three outcomes. In contrast, CSE was associated only with psychosomatic symptoms, and RESE‐W showed no significant associations.
• Study 1 informed the selection of an intervention target and clinical context for an exploratory unit‐tailored programme, illustrating a preliminary diagnostic‐to‐intervention logic.
• Interaction‐focused self‐efficacy increased immediately after the programme; controlled studies with longer follow‐up are needed to examine causal attribution, durability and downstream outcomes.

## 1. Introduction

Healthcare workers are increasingly exposed to disrespectful behaviours from patients, relatives and visitors, collectively referred to as ‘users’ to capture the heterogeneity of public healthcare interactions [1–5]. Among these behaviours, user incivility—low‐intensity deviant behaviour with ambiguous intent to harm that violates norms of mutual respect [6]—is particularly pervasive. When enacted by users, such norm violations concern broader expectations of interpersonal respect and dignity rather than internal organisational norms [7, 8]. Even when individual episodes appear minor, recurrent incivility may consume attention, emotional regulation and relational effort, while threatening dignity, perceived competence and emotional energy [8–10]. Conservation of resources (COR) theory proposes that stress arises when valued resources are threatened or lost, or when resource investment fails to yield the expected return [11, 12]. Repeated incivility may therefore contribute to both psychosomatic symptoms, as a health‐related manifestation of accumulated strain, and lower job satisfaction, as workers derive fewer valued returns—such as accomplishment, recognition and meaning—from their work [13–15].

Social cognitive theory (SCT) and COR theory offer complementary perspectives on self‐efficacy as a potentially relevant resource. SCT defines self‐efficacy as the perceived capability to organise and execute courses of action and explains how such beliefs shape appraisal, self‐regulation and behaviour [16–18]. From a COR perspective, efficacy beliefs may help workers mobilise knowledge, coping strategies and available support when demands arise [11, 19]. However, their usefulness depends on the capability assessed and on contextual conditions that enable perceived capability to be translated into action [11, 16, 20]. Self‐efficacy is therefore not a context‐free sense of confidence: Its predictive relevance should increase when the capability judgement aligns with the focal demand or outcome, as SCT’s correspondence principle states [19, 21]. This may explain why general self‐efficacy has not consistently buffered the consequences of workplace mistreatment among healthcare workers and why more specific efficacy beliefs warrant examination [2, 10].

Accordingly, the present study focuses on three efficacy beliefs that differ in their level of specificity and primary capability domain. Trauma‐related coping self‐efficacy (CSE) is an event‐ and recovery‐specific belief about perceived capability to manage personal functioning and cope with the internal and external demands associated with highly distressing experiences [5, 22, 23].

Regulatory emotional self‐efficacy in managing negative emotions at work (RESE‐W) is a domain‐specific belief about perceived capability to regulate states such as anger, irritation, distress and discouragement [24–27]. Self‐efficacy in managing challenging user behaviour is an interaction‐ and task‐specific belief about perceived capability to manage the encounter itself by recognising warning signs, remaining calm, communicating effectively, setting boundaries, using de‐escalation and safety strategies and requesting assistance [2, 4, 28–31]. These beliefs may overlap, because directly managing a challenging encounter can require emotional regulation and subsequent coping. Their primary referents nevertheless differ: CSE concerns coping with the consequences of a distressing encounter; RESE‐W concerns regulating negative emotions across work situations; and self‐efficacy in managing challenging user behaviour concerns the direct behavioural and interpersonal management of the encounter.

These distinctions suggest differentiated yet nonexclusive associations with the study outcomes. Interaction‐ and task‐specific self‐efficacy may be particularly relevant to perceived user incivility, as it concerns how ambiguous cues and emerging tension are appraised and managed during the encounter [2, 31, 32]. CSE and RESE‐W may be especially relevant to psychosomatic symptoms, as they relate to recovery from distressing experiences and the regulation of negative emotional activation, although effective interaction management may also limit resource depletion and strain [11, 25, 33]. Job satisfaction provides a broader positive occupational criterion: Efficacy beliefs may help preserve it by limiting resource loss and sustaining mastery, professional competence and access to valued aspects of care work [16, 29]. Rather than assuming a strict one‐to‐one correspondence between each efficacy belief and a single outcome, the study examines whether their distinct functional foci align with more or less consistent prospective patterns across perceived user incivility, psychosomatic symptoms and job satisfaction.

Identifying the efficacy belief with the most consistent pattern also has implications for intervention design. Self‐efficacy beliefs are not inherently fixed; their relative stability and responsiveness to experience may vary by specificity and the learning opportunities available in the relevant context. They can be strengthened through mastery experiences, vicarious learning, social persuasion and the interpretation of physiological and affective states [16, 21], but may also be weakened by adverse work experiences [34]. Daily diary evidence suggests that user incivility can deplete self‐efficacy and other personal resources [14, 35]. From a COR perspective, targeted training may support resource preservation and development by providing relevant practice, feedback, knowledge and social support, although it should complement rather than replace adequate organisational resources [11]. Healthcare programmes have used simulation, communication and aggression‐management strategies to strengthen professionals’ confidence in handling difficult interactions [28, 30, 36–40]. However, such interventions often adopt broad approaches or focus predominantly on high‐intensity aggression without first identifying the efficacy belief most relevant to recurrent, lower intensity incivility [2, 41, 42].

The present research addresses these limitations through two linked studies. Study 1 examines which of the three efficacy beliefs shows the most consistent prospective pattern across baseline‐adjusted changes in perceived user incivility, psychosomatic symptoms and job satisfaction over 3 months. Study 2 uses this diagnostic evidence to select the target of an exploratory, unit‐tailored training programme and examines its immediate pre–post change. Because communication behaviours provide practical means through which perceived capability may be enacted and were directly addressed by the programme, communication competencies are also examined as secondary, exploratory outcomes [32, 43–45]. Together, the studies follow a diagnostic‐to‐intervention logic linking resource identification to context‐sensitive preventive action in acute clinical settings.

## 2. Study 1: Identifying the Self‐Efficacy Belief Most Consistently Associated With Change

### 2.1. Aim of Study 1

Study 1 aimed to examine which of three functionally distinct self‐efficacy beliefs showed the most consistent prospective pattern of associations with baseline‐adjusted changes in healthcare workers’ perceived exposure to user incivility, psychosomatic symptoms and job satisfaction over 3 months. Specifically, Study 1 examined trauma‐related CSE, RESE‐W and self‐efficacy in managing challenging user behaviour. Study 1 was reported following the Strengthening the Reporting of Observational Studies in Epidemiology (STROBE) guidelines [46, 47] (see Supporting Information S1). Figure 1 presents the conceptual and analytical relationships examined.

**FIGURE 1:**
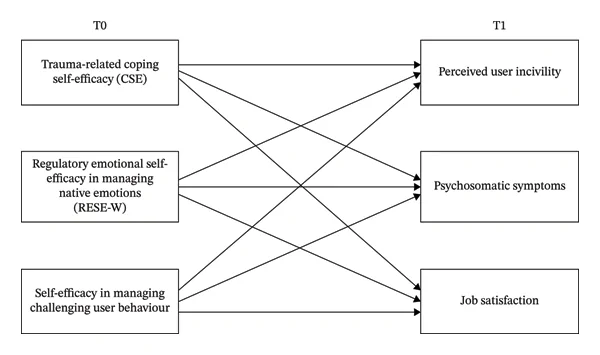
Conceptual and analytical framework of Study 1. *Note.* Arrows represent prospective associations examined in separately estimated regression models controlling for sex and job tenure; they do not imply causal effects. Outcome boxes represent standardised residualised change from baseline (T0) to the 3‐month follow‐up (T1).

### 2.2. Method

#### 2.2.1. Design and Participants

Study 1 was conducted in a healthcare facility in northern Italy using a two‐wave design with a 3‐month interval. The study examined whether baseline self‐efficacy beliefs were associated with subsequent changes in perceived user incivility, psychosomatic symptoms and job satisfaction. The research complied with the ethical standards of the Italian National Psychological Association and received approval from the Ethics Committee of University of Pavia (Protocol Code: 175/24, approved 10 June 2024). All participants provided informed consent before completing the survey.

Eligible participants were healthcare and social care professionals employed at the participating facility whose roles involved direct contact with users, including patients, relatives and visitors. Recruitment followed an organisationally mediated, nonprobability, voluntary‐response procedure. The research team prepared a formal communication outlining the study objectives and data collection procedures, which was first shared with departmental management and then disseminated to eligible professionals through unit coordinators and hospital communication channels. Participation was voluntary, and data were collected via an anonymous online questionnaire that took approximately 15 min to complete.

We disseminated the invitation to the accessible population of eligible professionals rather than to a predetermined random sample. Because we recruited through organisational communication channels and did not track individual receipt or viewing of the invitation, we could not determine the exact number of eligible professionals who received it. Consequently, we could not calculate a conventional response rate.

We did not conduct a formal a priori power analysis or sample‐size calculation. We determined the longitudinal sample size based on voluntary participation and the availability of sufficient matched data across both assessment waves. Given the fixed analytic samples, we conducted a sensitivity power analysis using G∗Power 3.1. For fixed‐model multiple regression tests of *R* ^2^ increase, with one tested predictor, three total predictors, *α* = 0.05 and 80% power, the model‐specific sample sizes of 72–74 yielded minimum detectable incremental effects ranging from *f* ^2^ = 0.109 to *f* ^2^ = 0.112. Study 1 was therefore sensitive to incremental effects of approximately *f* ^2^ = 0.11 or larger.

A total of 125 healthcare workers initially participated. Of these, 74 (59.2%) provided sufficient matched data across both assessment waves and were retained in the longitudinal analyses, whereas 51 (40.8%) were not retained. We retained participants who completed both assessment waves and provided at least 60% of the required survey data across the two‐wave protocol; all other participants were classified as not retained. The overall nonretention rate was therefore 40.8%. To assess potential attrition‐related selection bias, we compared retained and nonretained participants on available baseline sociodemographic, occupational and focal study variables. No statistically significant differences were observed in sex assigned at birth, job tenure, percentage of working time spent in direct contact with users, self‐efficacy in managing challenging user behaviour, trauma‐related CSE, RESE, perceived user incivility, psychosomatic symptoms or job satisfaction (all *p*s > 0.05). These analyses did not indicate systematic nonretention with respect to the observed baseline characteristics. However, they cannot definitively establish the missing‐data mechanism or exclude differences related to unmeasured characteristics or unobserved values.

The final sample consisted of 74 participants. The sample was composed predominantly of nurses (71.2%), followed by physicians (12.3%) and other healthcare professionals (16.4%; e.g., technicians, healthcare assistants). The majority were women (81.1%). Participants reported an average of 15.82 years of job tenure (SD = 10.52; range = 1–40), and they spent an average of 82.73% (SD = 19.42) of their working time in direct contact with users.

#### 2.2.2. Measures

All self‐efficacy constructs were assessed at baseline (T0). Perceived user incivility, psychosomatic symptoms and job satisfaction were measured at both T0 and follow‐up (T1), with a 3‐month interval between waves.

*Self-efficacy in managing challenging user behaviour* was assessed using the 10‐item *Confidence in Coping with Patient Aggression Instrument* (CCPAI) [31]. Although originally developed to assess self‐efficacy in coping with patient aggression, the instrument was used in the present study to assess perceived capability to manage emotionally tense or challenging user behaviours, including low‐intensity interpersonal demands relevant to incivility (e.g., ‘How self‐assured do you feel in the presence of a challenging patient?’). Participants responded with reference to their general experiences with challenging user interactions on a 5‐point Likert scale (1 = *strongly disagree*, 5 = *strongly agree*), with higher scores indicating greater perceived capability.

*Trauma-related CSE* was assessed using the six‐item *Trauma-Related Coping Self-Efficacy Scale* [5]. Participants indicated the extent to which they felt capable of managing the emotional and cognitive impact of challenging user behaviours (e.g., ‘Dealing with distressing images or memories related to the incident’) on a 7‐point scale (1 = *completely incapable*, 7 = *fully capable*). Participants were instructed to refer to a specific recent episode of incivility or aggression when responding.

*RESE-W* was measured using six items from the *Regulatory Emotional Self-Efficacy* scale for negative emotions, adapted for organisational settings [24], e.g., ‘remaining calm during stressful events or work‐related frustrations’. Responses were provided with reference to participants’ general work experiences on a 5‐point scale (1 = *strongly disagree*, 5 = *strongly agree*).

*Perceived user incivility* was measured using the four‐item short version of the *Customer Incivility Scale*, adapted for the Italian hospital context [48]. Participants indicated how frequently they experienced uncivil behaviours from users over the past 3 months (e.g., ‘Users directed rude or offensive comments at me’) on a five‐point Likert scale (1 = *never*, 5 = *always*).

*Psychosomatic symptoms* were assessed using the corresponding subscale of the Italian version of the short form of the *Burnout Assessment Tool* (BAT) [49]. This five‐item scale captures the frequency of physical stress‐related complaints (e.g., ‘I suffer from headaches’) on a five‐point Likert scale (1 = *never*, 5 = *always*).

*Job satisfaction* was assessed with a single‐item measure [50]: ‘How satisfied have you been with your work?’ rated on a 10‐point scale (0 = *not at all satisfied* to 10 = *completely satisfied*). Because job satisfaction was assessed using a single item, an internal consistency coefficient was not applicable. Nevertheless, meta‐analytic evidence supports the reliability and convergent validity of single‐item overall job satisfaction measures relative to multi‐item scales [27, 51].

#### 2.2.3. Data Analysis

All statistical analyses were performed using IBM SPSS Statistics (Version 28.0). The analytic strategy was designed to distinguish mean‐level change over time from interindividual differences in change. First, we calculated means, standard deviations (SDs) and sample‐ and occasion‐specific Cronbach’s alpha coefficients for the multi‐item measures, as well as zero‐order associations among the study variables. Pearson’s *r* or Spearman’s rho was used as indicated in Table 1. These analyses described the relationships among the focal self‐efficacy beliefs, outcomes and control variables and provided an initial indication of temporal stability.

**TABLE 1:** Descriptive statistics and intercorrelations among Study 1 variables.

Variable	*M*	SD	Skew.	Kurt.	1	2	3	4	5	6	7	8	9	10	11
1. Sex	—	—	—	—	—										
2. Job tenure	15.82	10.52	—	—	0.269^a∗^	—									
3. Self‐efficacy_T0	3.55	0.61	−0.13	−0.14	−0.257^a^	−0.113^a^	**0.850**								
4. CSE_T0	5.67	1.37	−1.02	0.76	−0.013^a^	0.100^b^	0.267^a∗^	**0.935**							
5. RESE‐W_T0	3.69	0.78	−0.57	0.22	0.001^a^	0.113^b^	0.279^a∗^	0.462^a∗∗^	**0.909**						
6. Incivility_T0	1.01	0.97	0.87	−0.23	0.130^a^	−0.331^b∗∗^	−0.076^a^	−0.102^a^	−0.187^a^	**0.903**					
7. Psychosomatic symptoms_T0	1.17	0.92	0.39	−0.89	−0.002^a^	−0.189^b^	−0.136^a^	−0.245^a∗^	−0.322^a∗∗^	0.460^a∗∗^	**0.875**				
8. Job satisfaction_T0	6.92	1.91	−0.89	0.57	−0.113^a^	0.129^b^	0.303^a∗∗^	0.329^a∗∗^	0.394^a∗∗^	−0.194^a^	−0.158^a^	—			
9. Incivility_T1	0.97	0.98	0.80	−0.46	0.036^a^	−0.239^b∗^	−0.268^a∗^	−0.099^a^	−0.276^a∗^	0.623^a∗∗^	0.361^a∗∗^	−0.209^a^	**0.913**		
10. Psychosomatic symptoms_T1	0.94	0.98	0.98	0.27	0.156^a^	−0.195^b^	−0.292^a∗^	−0.372^a∗∗^	−0.326^a∗∗^	0.262^a∗^	0.337^a∗∗^	−0.249^a∗^	0.385^a∗∗^	**0.930**	
11. Job satisfaction_T1	6.80	1.84	−0.88	0.88	−0.110^a^	0.148^b^	0.437^a∗∗^	0.170^a^	0.290^a∗^	−0.226^a^	−0.024^a^	0.482^a∗∗^	−0.400^a∗∗^	−0.377^a∗∗^	—

*Note: M* = mean; SD = standard deviation; Skew. = skewness; Kurt. = kurtosis. Sex: 0 = man, 1 = woman; *T*0 = baseline; *T*1 = 3‐month follow‐up; job tenure is reported in years; self‐efficacy = self‐efficacy in managing challenging user behaviour; CSE = trauma‐related coping self‐efficacy; RESE‐W= regulatory emotional self‐efficacy in managing negative emotions at work; incivility = perceived user incivility. Diagonal values represent internal consistency reliability (Cronbach’s *α*) for multi‐item scales. Superscript *a* indicates Pearson correlation coefficients; superscript *b* indicates Spearman’s rho coefficients. The bold values on the diagonal represent Cronbach’s alpha coefficients for internal consistency reliability.

^∗^ *p* < 0.05, ^∗∗^ *p* < 0.01.

To examine the empirical distinctiveness of the three self‐efficacy measures, we also calculated point estimates of the heterotrait–monotrait (HTMT) ratio of correlations [52] using absolute item‐level Pearson correlations. HTMT compares the average correlations between indicators belonging to different constructs with the geometric mean of the average correlations among indicators belonging to the same constructs. Values below the conservative 0.85 criterion were interpreted as indicating no substantive discriminant validity concern. Because the estimates were obtained in a modest sample and bootstrap confidence intervals were not calculated, we interpreted them as supportive rather than conclusive evidence of discriminant validity.

Second, we conducted paired‐samples *t* tests to examine whether perceived user incivility, psychosomatic symptoms and job satisfaction changed between T0 and T1. We also calculated Pearson correlations between T0 and T1 to assess temporal stability.

Third, to identify individual characteristics associated with change over time, we computed standardised residualised change scores for each outcome. Specifically, we regressed each T1 outcome on its corresponding T0 score, saved the resulting standardised residuals and used them as indicators of change. This approach captures the extent to which individuals’ follow‐up scores are higher or lower than expected given their baseline levels and provides a baseline‐adjusted indicator of relative change across two measurement occasions [53–55]. The residualised change approach was selected because the primary objective was to examine whether baseline efficacy beliefs were associated with individual differences in baseline‐adjusted follow‐up outcomes, rather than to model developmental trajectories. It therefore provided a direct, parsimonious outcome that could be interpreted consistently across the three domains. Alternative longitudinal approaches were considered. Mixed‐effects models are a valid alternative and could test a closely related question via a time‐by‐baseline–self‐efficacy interaction while retaining partially observed repeated outcomes under appropriate missing‐data assumptions [56, 57]. However, with only two assessment occasions, they would not provide a richer characterisation of individual trajectories. Latent change‐score models can explicitly account for measurement error. However, the modest sample, the two‐wave design and the single‐item assessment of job satisfaction did not support comparable latent analyses across the three outcomes [53, 58]. Cross‐lagged models were not applicable because the focal self‐efficacy beliefs were assessed only at baseline, precluding estimation of reciprocal longitudinal associations between self‐efficacy and the outcomes [59].

The standardised residualised change scores were then used as dependent variables in multiple regression models. For perceived user incivility and psychosomatic symptoms, lower residualised scores indicated more favourable change; for job satisfaction, higher residualised scores indicated more favourable change. Separate models were estimated for each outcome and each focal efficacy belief, controlling for sex and job tenure. Self‐efficacy in managing challenging user behaviour was entered as the focal predictor in the primary models and replaced by CSE or RESE‐W in parallel models. Because the three efficacy beliefs were examined in separately estimated models rather than entered simultaneously, the analyses permitted comparison of the consistency of their predictive patterns but did not test their unique relative contributions.

### 2.3. Results

#### 2.3.1. Descriptive Analyses

Table 1 reports means, SDs and zero‐order correlations among the study variables. All multi‐item measures demonstrated good to excellent internal consistency in the present sample, with Cronbach’s *α* coefficients ranging from 0.85 to 0.94 across constructs and assessment occasions (see Table 1).

Self‐efficacy in managing challenging user behaviour was positively associated with subsequent job satisfaction (*r* = 0.44, *p* < 0.01) and negatively associated with subsequent perceived incivility (*r* = −0.27, *p* < 0.05) and psychosomatic symptoms (*r* = −0.29, *p* < 0.05), indicating a favourable pattern of associations. Baseline CSE and RESE‐W showed a broadly similar correlational pattern, with small‐to‐moderate negative associations with psychosomatic symptoms (rs = −0.25 to −0.37) and positive associations with job satisfaction (rs = 0.17 to 0.39). Perceived incivility and psychosomatic symptoms were moderately to strongly correlated both within and across time points (rs = 0.26–0.46).

Temporal stability was moderate for perceived incivility (*r* = 0.62, *p* < 0.001) and job satisfaction (*r* = 0.48, *p* < 0.001), and lower for psychosomatic symptoms (*r* = 0.34, *p* = 0.003), indicating both continuity and meaningful interindividual variability over time. Job tenure showed small negative correlations with perceived incivility at both time points (rs = −0.33 to −0.24), suggesting that workers with longer tenure tended to report lower exposure to uncivil user behaviour.

The three efficacy beliefs were positively but only modestly to moderately intercorrelated at the composite‐score level. Self‐efficacy in managing challenging user behaviour was correlated with CSE (*r* = 0.27, *p* < 0.05) and RESE‐W (*r* = 0.28, *p* < 0.05), whereas CSE and RESE‐W showed a comparatively stronger association (*r* = 0.46, *p* < 0.01). To examine their empirical distinctiveness at the item level, HTMT ratios were also calculated. HTMT values were 0.512 for CSE and RESE‐W, 0.386 for RESE‐W and self‐efficacy in managing challenging user behaviour, and 0.315 for CSE and self‐efficacy in managing challenging user behaviour. All values were well below the conservative 0.85 criterion, providing supportive evidence that the three measures captured related but empirically distinguishable efficacy beliefs.

#### 2.3.2. Mean‐Level Change From T0 to T1

To distinguish whether the study variables changed over time from what predicted interindividual variability in change, we first examined mean‐level change using paired‐samples *t* tests. We then tested predictors of change in separate regression models using standardised, residualised change scores.

Perceived user incivility remained stable over time, *t* (73) = 0.41, *p* = 0.681, with moderately high temporal stability between T0 and T1 (*r* = 0.62, *p* < 0.001). Mean levels were very similar at T0 (*M* = 1.01) and T1 (*M* = 0.97). Raw change scores nevertheless varied substantially (Δ range = −2.25 to 2.75; *M* = −0.04; SD = 0.85), indicating meaningful between‐person variation in change.

Psychosomatic symptoms showed a small but nonsignificant decrease from T0 to T1, *t* (73) = 1.82, *p* = 0.066, with low‐to‐moderate temporal stability, *r* = 0.34, *p* = 0.003. Mean levels declined from T0 (*M* = 1.17) to T1 (*M* = 0.94), but this reduction did not reach conventional statistical significance. Again, substantial interindividual variability was observed in raw change scores (Δ range = −3.50 to 3.00, *M* = −0.23, SD = 1.10).

Job satisfaction also remained stable over time, *t* (73) = 0.54, *p* = 0.588, and showed moderate temporal stability between T0 and T1, *r* = 0.48, *p* < 0.001. Mean levels were similar at T0 (*M* = 6.92) and T1 (*M* = 6.80), although raw change scores revealed broad individual differences (Δ range = −5.00 to 5.00, *M* = −0.12, SD = 1.09).

Overall, none of the three outcomes showed significant mean‐level change over time. However, the dispersion of individual change scores indicated sufficient between‐person variability to examine whether baseline self‐efficacy beliefs were associated with baseline‐adjusted relative change.

#### 2.3.3. Regression Models Predicting Standardised Residualised Change

##### 2.3.3.1. Self‐Efficacy in Managing Challenging User Behaviour

As shown in Table 2, self‐efficacy in managing challenging user behaviour showed the most consistent pattern of significant associations with standardised residualised change across the three outcomes. For perceived user incivility, the overall model did not reach conventional statistical significance, *F* (3, 70) = 2.59, *p* = 0.060, *R* ^2^ = 0.100. Within this nonsignificant overall model, the focal coefficient for self‐efficacy in managing challenging user behaviour was significant: Higher self‐efficacy was associated with lower residualised change in perceived incivility, *B* = −0.526, SE = 0.192, *β* = −0.321, *p* = 0.008. Sex and job tenure were not significant predictors.

**TABLE 2:** Results of multiple regression analyses in study 1 predicting standardised residualised change in perceived user incivility (Model 1), psychosomatic symptoms (Model 2) and job satisfaction (Model 3) from baseline self‐efficacy in managing challenging user behaviour.

Predictor	Model 1: Perceived user incivility			Model 2: Psychosomatic symptoms			Model 3: Job satisfaction		
	*B*	SE	*β*	*B*	SE	*β*	*B*	SE	*β*
Sex	−0.326	0.305	−0.129	0.380	0.304	0.151	−0.028	0.302	−0.011
Job tenure	−0.004	0.011	−0.038	−0.016	0.011	−0.173	0.013	0.011	0.141
Self‐efficacy in managing challenging user behaviour	−0.526	0.192	−0.321^∗∗^	−0.397	0.192	−0.242^∗^	0.568	0.199	0.333^∗∗^
*R* ^2^	0.100			0.107			0.120		
Adjusted *R* ^2^	0.061			0.068			0.082		
*F*	2.587			2.785^∗^			3.140^∗^		

*Note.* Sex was coded 0 = man, 1 = woman. Job tenure was reported in years. *B* = unstandardised regression coefficient; SE = standard error; *β* = standardised regression coefficient. Dependent variables were standardised residualised change scores, computed by regressing each T1 outcome on its corresponding T0 score and saving standardised residuals. Accordingly, baseline outcome levels were not entered separately in the regression models. Ns reflects listwise deletion: *n* = 74 for Models 1 and 2; *n* = 73 for Model 3.

^∗^ *p* < 0.05, ^∗∗^ *p* < 0.01.

For psychosomatic symptoms, the regression model was significant, *F* (3, 70) = 2.79, *p* = 0.047, *R* ^2^ = 0.107. Higher self‐efficacy in managing challenging user behaviour predicted lower residualised change in psychosomatic symptoms, *B* = −0.397, SE = 0.192, *β* = −0.242, *p* = 0.042, whereas sex and job tenure were again nonsignificant.

For job satisfaction, the model was also significant, *F* (3, 69) = 3.14, *p* = 0.031, *R* ^2^ = 0.120. Higher self‐efficacy in managing challenging user behaviour predicted more positive residualised change in job satisfaction, *B* = 0.568, SE = 0.199, *β* = 0.333, *p* = 0.006. Neither sex nor job tenure significantly contributed to the model.

##### 2.3.3.2. Trauma‐Related CSE

When baseline CSE was entered as the focal predictor (Table 3), a more circumscribed pattern emerged. The model predicting residualised change in perceived user incivility was not significant, *F* (3, 68) = 0.10, *p* = 0.963, *R* ^2^ = 0.004, and CSE was not associated with change in perceived incivility, *B* = −0.033, SE = 0.088, *β* = −0.045, *p* = 0.711.

**TABLE 3:** Results of multiple regression analyses in Study 1 predicting standardised residualised change in perceived user incivility (Model 1), psychosomatic symptoms (Model 2) and job satisfaction (Model 3) from baseline trauma‐related coping self‐efficacy (CSE).

Predictor	Model 1: Perceived user incivility			Model 2: Psychosomatic symptoms			Model 3: Job satisfaction		
	*B*	SE	*β*	*B*	SE	*β*	*B*	SE	*β*
Sex	−0.110	0.322	−0.043	0.546	0.297	0.213	−0.155	0.318	−0.060
Job tenure	−0.001	0.012	−0.009	−0.013	0.011	−0.135	0.012	0.012	0.130
CSE	−0.033	0.088	−0.045	−0.212	0.082	−0.293^∗^	−0.001	0.087	−0.002
*R* ^2^	0.004			0.144			0.017		
Adjusted *R* ^2^	−0.040			0.107			−0.027		
*F*	0.095			3.826^∗^			0.382		

*Note.* Sex was coded 0 = man, 1 = woman. Job tenure was reported in years. *B* = unstandardised regression coefficient; SE = standard error; *β* = standardised regression coefficient. Dependent variables were standardised residualised change scores, computed by regressing each T1 outcome on its corresponding T0 score and saving standardised residuals. Accordingly, baseline outcome levels were not entered separately in the regression models. All models were based on *n* = 72 because of listwise deletion.

^∗^ *p* < 0.05, ^∗∗^ *p* < 0.01.

By contrast, the model predicting residualised change in psychosomatic symptoms was significant, *F* (3, 68) = 3.83, *p* = 0.014, *R* ^2^ = 0.144. Higher baseline CSE predicted lower residualised change in psychosomatic symptoms, *B* = −0.212, SE = 0.082, *β* = −0.293, *p* = 0.011. Sex and job tenure were not significant.

The model predicting residualised change in job satisfaction was not significant, *F* (3, 68) = 0.38, *p* = 0.766, *R* ^2^ = 0.017, and CSE was unrelated to change in job satisfaction, *B* = −0.001, SE = 0.087, *β* = −0.002, *p* = 0.988.

##### 2.3.3.3. RESE‐W

No statistically significant focal association emerged for baseline RESE‐W (Table 4). The model predicting residualised change in perceived user incivility was not significant, *F* (3, 70) = 1.10, *p* = 0.356, *R* ^2^ = 0.045, and RESE‐W did not significantly predict change in perceived incivility, *B* = −0.261, SE = 0.150, *β* = −0.205, *p* = 0.087.

**TABLE 4:** Results of multiple regression analyses in Study 1 predicting standardised residualised change in perceived user incivility (Model 1), psychosomatic symptoms (Model 2) and job satisfaction (Model 3) from baseline regulatory emotional self‐efficacy for negative emotions (RESE‐W).

Predictor	Model 1: perceived user incivility			Model 2: psychosomatic symptoms			Model 3: job satisfaction		
	*B*	SE	*β*	*B*	SE	*β*	*B*	SE	*β*
Sex	−0.148	0.306	−0.059	0.508	0.297	0.202	−0.211	0.309	−0.084
Job tenure	0.001	0.012	0.007	−0.012	0.011	−0.130	0.010	0.012	0.103
RESE‐W	−0.261	0.150	−0.205	−0.272	0.146	−0.214	0.148	0.152	0.117
*R* ^2^	0.045			0.097			0.030		
Adjusted *R* ^2^	0.004			0.058			−0.012		
*F*	1.099			2.493			0.713		

*Note:* Sex was coded 0 = man, 1 = woman. Job tenure was reported in years. *B* = unstandardised regression coefficient; SE = standard error; *β* = standardised regression coefficient. Dependent variables were standardised residualised change scores, computed by regressing each T1 outcome on its corresponding T0 score and saving standardised residuals. Accordingly, baseline outcome levels were not entered separately in the regression models. Ns reflects listwise deletion: *n* = 74 for Models 1 and 2; *n* = 73 for Model 3.

^∗^ *p* < 0.05, ^∗∗^ *p* < 0.01.

Similarly, the model predicting residualised change in psychosomatic symptoms did not reach significance, *F* (3, 70) = 2.49, *p* = 0.067, *R* ^2^ = 0.097. RESE‐W showed a negative but nonsignificant association with residualised change in psychosomatic symptoms, *B* = −0.272, SE = 0.146, *β* = −0.214, *p* = 0.067.

Finally, the model predicting residualised change in job satisfaction was not significant, *F* (3, 69) = 0.71, *p* = 0.548, *R* ^2^ = 0.030, and RESE‐W was unrelated to change in job satisfaction, *B* = 0.148, SE = 0.152, *β* = 0.117, *p* = 0.334.

Figure 2 provides a graphical summary of the significant and nonsignificant predictive associations observed in Study 1.

**FIGURE 2:**
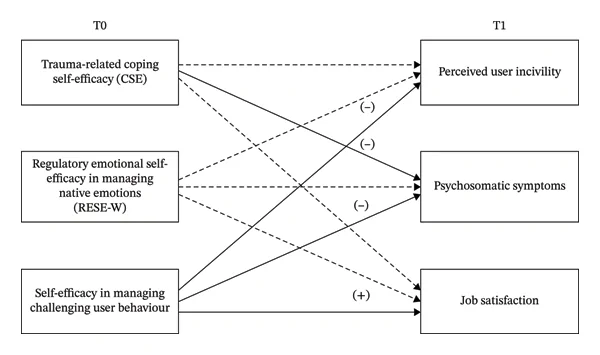
Summary of the predictive associations observed in Study 1. *Note.* Solid arrows indicate statistically significant focal associations, whereas dashed arrows indicate nonsignificant associations. Plus and minus signs indicate the direction of statistically significant associations. Each efficacy belief was examined in a separately estimated regression model controlling for sex and job tenure; arrows do not imply causal effects.

### 2.4. Summary of Study 1 and Rationale for Study 2

Taken together, Study 1 indicated that self‐efficacy in managing challenging user behaviour showed the most consistent predictive pattern across outcomes. It showed statistically significant focal coefficients for standardised residualised change in all three outcomes, although the overall model predicting perceived user incivility did not reach conventional significance. Higher levels were associated with lower‐than‐expected follow‐up levels of perceived incivility and psychosomatic symptoms and higher‐than‐expected follow‐up levels of job satisfaction after accounting for baseline outcome levels.

By contrast, baseline CSE showed a narrower pattern of associations, significantly predicting only lower residualised change in psychosomatic symptoms. Baseline RESE‐W did not significantly predict standardised residualised change in any of the three outcomes, although its associations with perceived incivility and psychosomatic symptoms were in the expected direction. Because the three efficacy beliefs were examined in separate models, these results do not establish that self‐efficacy in managing challenging user behaviour made a uniquely stronger contribution than CSE or RESE‐W. Rather, it showed the most consistent pattern across the outcomes examined and was therefore selected as the most empirically supported candidate for the exploratory intervention in Study 2.

Beyond the primary focus on self‐efficacy, Study 2 also examined communication competencies as secondary outcomes. This choice reflected the conceptual link between perceived capability to manage challenging user behaviour and the interpersonal skills that can support managing such situations in practice. While self‐efficacy concerns professionals’ beliefs in their capability to manage challenging interactions, the communication outcomes assessed self‐reported communication competencies and behaviours. Clear information provision, empathy, respectful communication and assertive interpersonal behaviour may help professionals recognise and respond to users’ needs, reduce misunderstanding, establish appropriate boundaries and prevent or de‐escalate interpersonal tension [43, 60–62]. Consistent with SCT [16, 21], practising these skills may also provide mastery experiences that strengthen perceived capability, while stronger efficacy beliefs may support their effective enactment during demanding encounters. Previous research indicates that communication and aggression‐management training can improve both healthcare professionals’ communication performance and their confidence or self‐efficacy in managing specific interpersonal demands [28, 32, 44, 45]. Directional short‐term improvements in communication outcomes were therefore expected because the training directly addressed these competencies. However, the study did not specify a quantitative effect size or minimum meaningful degree of change a priori and treated the communication outcomes as secondary and exploratory. Their relationship with self‐efficacy was theoretically proposed but was not tested as a mediating mechanism.

## 3. Study 2: Exploratory Targeted Training to Strengthen the Selected Efficacy Belief

### 3.1. Aim of Study 2

Based on the findings of Study 1, Study 2 aimed to explore whether self‐efficacy in managing challenging user behaviour could be strengthened through a brief, context‐sensitive, unit‐tailored training programme delivered in acute clinical settings. A secondary, exploratory aim was to examine whether the intervention was also associated with improvements in communication competencies relevant to managing challenging user interactions. We treated self‐efficacy in managing challenging user behaviour as the primary outcome, whereas respect and authenticity, empathy, informative communication and social skills were treated as secondary, exploratory outcomes. Because the communication outcomes were secondary and exploratory, we anticipated immediate directional improvements because the corresponding competencies were directly addressed during the programme; however, we did not establish a specific quantitative effect‐size threshold or minimum meaningful change a priori. We included these outcomes as self‐reported indicators of broader communication behaviours relevant to challenging interactions, rather than as objective measures of behavioural performance or proposed mediators of change in self‐efficacy. Consistent with this exploratory objective, Study 2 was not designed to establish definitive intervention effectiveness, but rather to examine immediate within‐group change, implementation in acute clinical settings, and participant‐rated acceptability. Study 2 was reported in accordance with the Transparent Reporting of Evaluations with Nonrandomized Designs (TREND) statement and the Template for Intervention Description and Replication (TIDieR) checklist [63, 64]; see Supporting Information S2, S3).

### 3.2. Method

#### 3.2.1. Design and Participants

Study 2 was conducted within the same healthcare facility and under the same ethics‐approved research project (Protocol Code: 175/24). A single‐group pre–post design was used, with the postintervention assessment administered immediately after the final training session. All participants provided informed consent before the preintervention assessment.

Study 2 used a two‐stage, nonprobability sampling procedure. At the first stage, nurses were purposively selected as the target professional group because their work involves sustained, direct interaction with patients and relatives, placing them in frequent contact with the interpersonal demands addressed by the programme [65, 66]. The Emergency Department and Neurology Unit were selected using a theory‐ and context‐informed procedure. Preliminary descriptive findings from Study 1 served as an exploratory screening tool and indicated comparatively higher levels of perceived user incivility and strain among participants working in these units. This information was considered alongside their clinical characteristics, including frequent acute interactions, time pressure and emotionally demanding encounters. The descriptive screening was used solely to inform intervention targeting and was not treated as an inferential comparison demonstrating statistically higher risk in these units. At the second stage, nurses employed in the selected units whose work involved direct contact with patients or relatives were eligible to participate. Eligible nurses were informed about the intervention through unit‐level organisational channels, and participation was voluntary. 22 enrolled nurses participated in the training; 21 completed both the preintervention and immediate postintervention assessments. Because recruitment was organised at the unit level and individual receipt of the invitation was not tracked against a complete invitation list, the exact denominator of invited nurses could not be reliably reconstructed, and a conventional participation rate could not be calculated.

No formal a priori power analysis or sample‐size calculation was conducted. The achieved sample was determined by the purposive selection of the clinical units, the accessible eligible nursing population and voluntary participation. Given the fixed sample of 21 complete pairs, a sensitivity power analysis was conducted using G∗Power 3.1. For a two‐tailed paired‐samples *t* test with *α* = 0.05 and 80% power, the minimum detectable within‐person effect was approximately *d*_*z*_ = 0.64. The study was therefore primarily sensitive to moderate‐to‐large immediate pre–post effects and had limited sensitivity to smaller changes, particularly in the secondary communication outcomes. Although the same sensitivity threshold applied mathematically to each paired comparison, the four communication outcomes were interpreted as exploratory rather than as co‐primary confirmatory outcomes. Because Study 1 data were collected anonymously, it was not possible to determine whether individual Study 2 participants had also participated in Study 1. The studies were therefore linked at the design level: Study 1 informed the selection of the intervention target and clinical context in Study 2, but the two studies did not constitute a confirmed longitudinal follow‐up of the same individuals.

#### 3.2.2. Intervention

The intervention consisted of a structured, evidence‐informed training programme delivered on‐site and tailored to the clinical realities of the Emergency Department and Neurology units. Although specific examples and scenarios were adapted to each service, the pedagogical architecture, learning objectives and core psychological principles remained consistent across settings. Training was conducted in small groups over a single day. Each module used unit‐specific clinical scenarios to ensure ecological validity and immediate relevance to practice. The facilitator provided structured debriefing and feedback after the experiential activities to support reflection, consolidate learning and reinforce perceived capability. A specialist in work and health psychology, with expertise in conflict management, de‐escalation and healthcare communication, delivered the programme.

The primary aim of the intervention was to strengthen staff self‐efficacy in managing challenging user behaviour. To do so, the programme combined skills‐based instruction, reflective practice and experiential exercises designed to enhance participants’ perceived capability to prevent escalation, intervene effectively during high‐tension encounters and recover adaptively after complex interactions.

Three interrelated competency domains were targeted: relational competencies, de‐escalation skills and assertive communication. Relational competencies included principles of trust‐building, benevolence, procedural justice, clarity and perspective‐taking, which were intended to support the prevention of escalation and strengthen the therapeutic relationship [67–69]. De‐escalation skills focused on identifying triggers of aggression, recognising early warning signs, managing the physical environment and using verbal and nonverbal techniques to reduce arousal in high‐tension situations [61, 68, 70]. Assertive communication strategies addressed boundary‐setting, negotiation and constructive conflict resolution, including first‐person statements, fact‐focused communication, verbal–nonverbal congruence and structured techniques such as the broken record, fogging, realistic compromise and assertive closure [62, 69].

In addition, the intervention incorporated supportive communication skills theoretically linked to self‐efficacy in managing challenging user behaviour. Informative communication was included to help professionals clearly convey expectations and boundaries, thereby reducing misunderstandings that may trigger rude behaviour [67, 71]. Empathy was targeted to support recognition of patients’ emotional states and facilitate tailored responses that de‐escalate tension [60]. Respect and authenticity were emphasised to reinforce trust and fairness and help prevent minor offences from escalating [60]. These communication domains were treated as behavioural resources that could support the enactment of perceived capability, while remaining conceptually distinct from self‐efficacy itself.

Finally, the programme included brief stress‐management and postevent recovery techniques such as controlled breathing, grounding strategies, cognitive distancing and reframing, postural regulation, progressive muscle relaxation, brief mindfulness practices and reflective journaling [72–74]. Together, the programme components were designed to engage the principal sources of efficacy proposed by SCT [16, 21]: Scenario‐based practice offered mastery experiences, peer observation supported vicarious learning, structured feedback and encouragement provided verbal persuasion, and stress‐management and reflective activities were intended to support more adaptive interpretation and regulation of physiological and affective activation.

#### 3.2.3. Measures

Self‐efficacy in managing challenging user behaviour and communication skills were assessed before and after the intervention, whereas training evaluation was assessed postintervention only. Postintervention measures were administered immediately upon completion of the training programme, at the end of the final training session.

*Self-efficacy in managing challenging user behaviour* was assessed using the same 10‐item CCPAI [31] described in Study 1 (*α*_pre_ = 75, *ɷ*_post_ = 75; *α*_pre_ = 87, *ɷ*_post_ = 89).

*Communication skills* were assessed using the 18‐item *Health Professionals’ Communication Skills Scale* (HP‐CSS [75]). The scale assesses four core domains of clinician–patient communication: informative communication (6 items; *α*_pre_ = 0.78, *ɷ*_post_ = 0.81; *α*_pre_ = 0.91, *ɷ*_post_ = 0.91), empathy (5 items; *α*_pre_ = 0.72, *ɷ*_post_ = 0.75; *α*_pre_ = 0.85, *ɷ*_post_ = 0.82), respect and authenticity (3 items; *α*_pre_ = 0.75, *ɷ*_post_ = 0.75; *α*_pre_ = 0.77, *ɷ*_post_ = 0.81) and social skills (4 items; *α*_pre_ = 0.73, *ɷ*_post_ = 0.76; *α*_pre_ = 0.86, *ɷ*_post_ = 0.88). Participants rated how frequently they displayed each behaviour on a 6‐point scale (1 = rarely, 6 = always), with higher scores indicating stronger self‐reported communication competence. Because the intervention explicitly targeted assertive communication, the HP‐CSS social skills domain was also considered, for exploratory interpretive purposes, as an indicator of assertiveness‐related communication. Accordingly, in the sections below, this outcome is referred to as assertiveness‐related communication. However, it derives from the broader HP‐CSS social skills subscale and should not be interpreted as a standalone or direct measure of assertiveness.

*Training evaluation* was assessed using two single‐item indicators developed for this study. Participants rated the perceived quality of the training content and the trainer’s delivery on a scale from 0 (*very poor*) to 10 (*excellent*).

#### 3.2.4. Data Analysis

All statistical analyses were performed using IBM SPSS Statistics (Version 28.0). The analyses focused on immediate within‐person change from preintervention to immediate postintervention rather than on estimating causal intervention effects. Paired‐samples *t* tests compared preintervention (T0) and postintervention (T1) scores across the targeted outcomes: self‐efficacy in managing challenging user behaviour, respect and authenticity, empathy, informative communication and assertiveness‐related communication (HP‐CSS social skills domain). For each variable, preintervention and immediate postintervention means and SDs, pre–post Pearson correlations (r), paired‐samples *t* tests and standardised within‐person effect sizes were computed. The pre–post correlations were used to describe rank‐order association across assessments and were not treated as indicators of mean‐level change. Descriptive statistics also summarised participants’ evaluations of the training content and delivery.

### 3.3. Results

#### 3.3.1. Pre–Post Changes

Paired‐samples *t* tests revealed a significant pre–post improvement in self‐efficacy in managing challenging user behaviour, the primary target of the intervention (Pre: *M* = 3.74, SD = 0.43; Post: *M* = 4.10, SD = 0.55; *t*_(20)_ = −3.54, *p* = 0.002; *d*_*z*_ = 0.77, see Table 5). The moderate pre–post correlation (*r* = 0.57, *p* = 0.007) indicated moderate rank‐order consistency across assessments.

**TABLE 5:** Pre‐ and immediate postintervention changes in self‐efficacy and communication competencies in Study 2.

Variable	*M* (pre)	SD (pre)	*M* (post)	SD (post)	*N*	*r*	*t* (df)	*p*
Self‐efficacy in managing challenging user behaviour	3.74	0.43	4.10	0.55	21	0.568^∗∗^	−3.54 (20)	0.002^∗∗^
Respect and authenticity	5.02	0.59	5.05	0.71	21	0.819^∗∗^	−0.36 (20)	0.724
Empathy	4.99	0.58	5.13	0.67	21	0.743^∗∗^	−1.38 (20)	0.183
Informative communication	5.07	0.57	5.24	0.65	21	0.748^∗∗^	−1.73 (20)	0.099
Social skills (assertiveness‐related communication)	4.46	0.68	4.63	0.77	21	0.668^∗∗^	−1.28 (20)	0.217

*Note: M* (Pre), SD (Pre) = Mean and standard deviation at the preintervention assessment; *M* (Post), SD (Post) = mean and standard deviation immediately after the final training session; *N* = number of participants with paired observations; *r* = Pearson product–moment correlation between pre‐ and postintervention scores; *t* (df) = test statistic and degrees of freedom from the paired‐samples *t* test; *p* = two‐tailed significance level.

^∗∗^ *p* < 0.01.

Exploratory, secondary communication outcomes—respect and authenticity, empathy, informative communication and assertiveness‐related communication (HP‐CSS social skills domain)—showed small, nonsignificant increases (all ps > 0.09). Participants also evaluated the training they received. The perceived quality of the intervention’s content was high (*M* = 9.00, SD = 1.11; range = 7–10), as was the perceived quality of the trainer’s delivery (*M* = 9.50, SD = 0.62; range = 8–10), indicating highly favourable evaluations of both training content and delivery.

### 3.4. Summary of Study 2

Taken together, Study 2 showed an immediate within‐group increase in self‐efficacy in managing challenging user behaviour following the brief, context‐sensitive training programme. Because the study used an uncontrolled single‐group design and an immediate post‐test, this result provides preliminary evidence of short‐term change in the targeted efficacy belief. By contrast, the exploratory secondary communication outcomes, including the HP‐CSS social skills domain used here as an indicator of assertiveness‐related communication, showed smaller, nonsignificant short‐term changes.

## 4. General Discussion

This paper reports two linked studies that connect resource identification to context‐sensitive preventive action in healthcare settings. Study 1 indicated that self‐efficacy in managing challenging user behaviour showed the most consistent prospective pattern across perceived user incivility, psychosomatic symptoms and job satisfaction. Building on this evidence, Study 2 showed an immediate increase in this efficacy belief following a brief, unit‐tailored training programme in acute nursing settings. Together, the two studies illustrate a diagnostic‐to‐intervention approach in which observational evidence is used to identify a potentially relevant psychological resource and inform an exploratory, context‐sensitive intervention. The findings should nevertheless be interpreted as prospective associations and immediate pre–post changes rather than as evidence of causal effects.

### 4.1. The Most Consistent Predictive Role of Task‐Specific Self‐Efficacy

Findings from Study 1 indicated differentiated prospective patterns across the three efficacy beliefs. Self‐efficacy in managing challenging user behaviour was significantly associated with standardised residualised change in all three outcomes: Higher baseline levels were associated with lower‐than‐expected follow‐up levels of perceived incivility and psychosomatic symptoms, and with higher‐than‐expected follow‐up levels of job satisfaction. The association with perceived incivility should, nevertheless, be interpreted cautiously because, although the focal coefficient was significant, the overall regression model did not reach conventional levels of statistical significance. CSE showed a more circumscribed pattern, being associated only with more favourable change in psychosomatic symptoms, whereas RESE‐W was not significantly associated with standardised residualised change in any outcome. These findings suggest that the relevance of an efficacy belief may depend on its functional alignment with the demands and outcomes under consideration, rather than indicating that one form of self‐efficacy is uniformly more beneficial than the others.

This differentiated pattern is consistent with the correspondence principle within SCT [16, 21]. CSE primarily concerns workers’ perceived capability to cope with the emotional, cognitive and situational consequences of a distressing experience [23, 33, 76, 77]. RESE‐W concerns perceived capability to regulate negative emotional states such as distress, frustration or tension [24, 26, 78]. By contrast, self‐efficacy in managing challenging user behaviour concerns the perceived ability to directly manage the encounter, including recognising warning signs, maintaining composure, communicating effectively, setting boundaries and using de‐escalation strategies [2, 31, 32, 61, 79]. Although these capability domains partially overlap, they differ in whether the efficacy judgement is centred primarily on postevent coping, emotional regulation or the behavioural and interpersonal management of the interaction.

The empirical relationships among the measures were consistent with this conceptual distinction. At the composite‐score level, the three efficacy beliefs were positively but only modestly to moderately intercorrelated (rs = 0.27–0.46). The item‐level HTMT ratio of correlations assessment provided further supportive evidence, with HTMT values ranging from 0.31 to 0.51 and therefore remaining well below the conservative 0.85 criterion [52]. The comparatively higher HTMT value between CSE and RESE‐W is consistent with their shared focus on managing internal responses to stressful or distressing experiences. The lower ratios involving self‐efficacy in managing challenging user behaviour are consistent with its additional behavioural and interpersonal focus. These findings therefore suggest that the three efficacy beliefs are related but empirically distinguishable rather than interchangeable.

The interaction‐focused nature of self‐efficacy in managing challenging user behaviour may help explain its broader pattern of associations. User incivility consists of ambiguous, low‐intensity behaviours that require professionals to appraise interpersonal cues, maintain communication clarity and regulate behaviour [6, 14, 15, 80]. From an SCT perspective [16], a strong belief in one’s capability to recognise escalation, remain composed, establish boundaries and respond assertively may support more constructive management of such encounters [4, 81]. These processes could contribute to lower perceived incivility over time by helping professionals interrupt emerging tension before it escalates. This interpretation remains tentative, however, because neither the behavioural strategies enacted by healthcare workers nor subsequent user responses were directly assessed.

The findings concerning psychosomatic symptoms can also be interpreted through COR [11]. CSE and RESE‐W may function as personal resources that support the regulation of internal responses to stress and limit resource depletion [5, 25, 27, 33], which is consistent with the association between CSE and more favourable change in psychosomatic symptoms and with the direction of the RESE‐W coefficients. Self‐efficacy in managing challenging user behaviour may additionally support resource preservation during the interaction itself. Successfully navigating challenging interactions may reduce further demands while fostering mastery, agency and professional competence, which could in turn support more favourable work‐related attitudes [16, 29, 82]. This provides one possible interpretation of its association with change in job satisfaction, although the proposed resource processes were not directly measured.

These findings do not establish that self‐efficacy in managing challenging user behaviour is intrinsically or uniquely superior to the other efficacy beliefs. The measures differed in their situational referents and response formats, and these methodological differences may have contributed to their observed intercorrelations and prospective associations. Moreover, the three efficacy beliefs were examined in separately estimated models rather than entered simultaneously, so their unique relative contributions were not directly tested. The findings should therefore be interpreted as showing that self‐efficacy in managing challenging user behaviour displayed the most consistent predictive pattern across the outcomes examined, rather than demonstrating its unique statistical superiority.

### 4.2. Short‐Term Changes Following the Intervention

Building on the findings from Study 1, Study 2 implemented an exploratory training programme to strengthen the efficacy belief that had shown the most consistent predictive pattern. Participants showed a significant short‐term increase in self‐efficacy in managing challenging user behaviour following the intervention. Because the postintervention assessment was administered immediately after the final training session and the study did not include a control group, this finding indicates an immediate pre–post change. However, it does not establish intervention effectiveness, durability or causal attribution.

The training architecture provides a theoretically plausible explanation for the observed change. Consistent with SCT [16], scenario‐based training offered opportunities for mastery experiences; observation of peers and structured feedback supported vicarious learning and social persuasion; and reflection on emotional and physiological responses encouraged participants to interpret arousal as manageable [28, 30, 45, 67, 79, 83]. These elements were combined with concrete strategies for recognising escalation cues, communicating assertively, setting boundaries and managing postevent activation. From a COR perspective [11, 12], the training may also be understood as one component of a resource‐supportive organisational passageway, providing knowledge, feedback, social learning and opportunities for skill rehearsal. Nevertheless, neither the proposed SCT mechanism nor the corresponding processes of resource preservation or gain were directly tested.

The exploratory communication outcomes showed only small, nonsignificant immediate changes. Directional improvements were anticipated because the programme addressed informative communication, empathy, respect and authenticity, and assertiveness‐related communication; however, no specific quantitative effect size or minimum meaningful degree of change was specified a priori. Moreover, the sensitivity analysis indicated that the modest Study 2 sample had limited ability to detect small‐to‐moderate changes. These nonsignificant findings should therefore be considered inconclusive rather than as evidence that communication did not change.

The different patterns across the two outcome types may also reflect their conceptual and measurement characteristics. Self‐efficacy is a cognitive judgement of perceived capability that can be updated relatively quickly through mastery, modelling and feedback [16, 21]. Communication competencies are broader behavioural repertoires that may require repeated practice, workplace reinforcement and opportunities for transfer before measurable change becomes apparent [28, 68–70]. The HP‐CSS domains were also assessed through self‐report and capture broad communication behaviours rather than the highly specific de‐escalation and boundary‐setting responses emphasised in the intervention. In particular, the social skills domain was used as an assertiveness‐related indicator rather than as a standalone measure of assertiveness. The immediate postintervention assessment may therefore have been more sensitive to changes in perceived capability than to durable changes in practice. The observed results may therefore indicate that the clearest immediate change was concentrated in the cognitive–evaluative component of managing challenging user behaviour, without demonstrating a corresponding short‐term change in broader self‐reported communication competencies. Overall, Study 2 shows that a brief, theoretically grounded programme can be delivered in acute clinical settings and can be followed by an immediate increase in self‐efficacy in managing challenging user behaviour. As an exploratory proof of concept, it provides a basis for controlled studies examining whether such changes persist and translate into observable communication behaviour, reduced exposure to user mistreatment and improved occupational well‐being.

### 4.3. Strengths, Limitations and Recommendations for Future Research

The present research has several strengths. Its sequential two‐study design links identifying the efficacy belief with the most consistent prospective pattern to an exploratory intervention targeting that belief. Grounded in SCT [16, 19], the study distinguishes among CSE, RESE‐W and self‐efficacy in managing challenging user behaviour, responding to calls for more task‐ and demand‐specific conceptualisations of self‐efficacy in healthcare stress research [2, 10]. The HTMT assessment provided additional evidence that the three measures captured empirically distinguishable efficacy beliefs. The two‐wave design also offered a temporal perspective on between‐person differences in baseline‐adjusted change, while the unit‐tailored, scenario‐based programme was theoretically coherent and ecologically grounded.

Despite these strengths, the study has several limitations that warrant caution when interpreting the findings. First, neither study was based on a formal a priori power analysis, and both used modest sample sizes. Sensitivity analyses indicated limited ability to detect smaller effects; consequently, nonsignificant findings (particularly those concerning the secondary communication outcomes) should be regarded as inconclusive rather than as evidence of no association or change. The modest samples may also have reduced the precision and stability of the estimates. Study 1 additionally experienced substantial nonretention. Although retained and nonretained participants did not differ significantly on observed baseline sociodemographic, occupational or focal study variables, these comparisons cannot establish that the data were missing completely at random or exclude selection related to unmeasured characteristics or unobserved values. Replication in larger samples, procedures to minimise attrition and controlled intervention designs with longer follow‐up are warranted.

Second, both studies were conducted within the same healthcare organisation in northern Italy. Their convergence should therefore be interpreted as evidence from a contextually linked research programme rather than as independent cross‐organisational replication. Organisational conditions—including staffing, workload, service organisation, managerial practices, available support and local interaction norms—may influence exposure to user incivility, workers’ responses, efficacy beliefs and training implementation [66, 84]. Broader cultural norms concerning acceptable interpersonal conduct, authority relationships, communication with healthcare professionals and expressions of dissatisfaction may likewise affect the occurrence, appraisal and reporting of incivility [7, 85]. Generalisability is further limited by nonprobability sampling, the inability to calculate conventional response or participation rates, predominantly female samples and the exclusive focus on nurses in Study 2. Future research should replicate the findings across organisations, regions, countries, professional groups and more demographically diverse samples, while examining organisational and cultural characteristics as potential moderators.

Third, Study 2 employed a single‐group pre–post design with no control group and only an immediate postintervention assessment. The observed change cannot therefore be attributed conclusively to the programme, and testing, expectancy, social desirability or temporary motivational effects cannot be excluded [86, 87]. Voluntary participation may also have attracted nurses with greater motivation, interest in the topic or readiness for change. These design choices reflected the operational constraints of implementing an exploratory training programme in acute clinical settings, where recruitment, randomisation and intervention implementation present practical challenges [88]. Future research should use controlled or randomised designs, document nonparticipant characteristics and include longer term follow‐up assessments to examine the durability and downstream effects of training.

Fourth, both studies relied primarily on self‐report measures. While self‐efficacy is inherently subjective and appropriately assessed through self‐report [89], perceptions of incivility and well‐being may be influenced by common method variance, recall processes or social desirability. The communication outcomes in Study 2 also captured broad self‐reported behaviours rather than directly observed performance and may therefore have been insufficiently sensitive to subtle, context‐specific behavioural change. Future research should incorporate behavioural observations, independently rated simulations, supervisor or peer reports, incident reports, patient complaints and other indicators of behavioural transfer.

Fifth, the two‐wave residualised change approach also has analytical limitations. Residualised scores provide a sample‐dependent measure of relative, baseline‐adjusted change and do not distinguish true change from measurement error within a latent‐variable framework. With only two assessment occasions, the design also cannot characterise nonlinear or more complex within‐person trajectories [53, 58]. Moreover, the primary complete‐case analyses did not use mixed‐effects or full‐information approaches to retain partially observed longitudinal outcomes under explicit missing‐data assumptions. The findings should therefore be interpreted as associations between baseline self‐efficacy and baseline‐adjusted relative follow‐up differences, rather than as estimates of individual developmental trajectories or causal effects. Future research should include larger samples, additional assessment waves, repeated measurement of both efficacy beliefs and outcomes, and more comprehensive longitudinal methods.

Finally, although the HTMT values supported the empirical differentiation of the three efficacy measures, the estimates were obtained in a modest sample. They should therefore be treated as supportive rather than definitive evidence of discriminant validity. Replication in larger samples using confirmatory measurement models, and more closely harmonised situational referents and response formats would provide a stronger assessment of construct distinctiveness.

### 4.4. Implications for Nursing Management

From a policy perspective, the results underscore the need to move beyond generic, one‐size‐fits‐all approaches to preventing the mistreatment of healthcare workers by users. Existing prevention approaches often prioritise high‐intensity physical aggression, potentially overlooking the cumulative impact of low‐intensity, recurrent user incivility [2, 4]. Healthcare organisations should therefore recognise incivility as a psychosocial risk factor and promote prevention strategies that target task‐specific psychological resources. Embedding support for self‐efficacy in managing challenging user behaviour within occupational health and safety frameworks may better align prevention policies with the day‐to‐day relational realities of clinical work [4, 16].

The findings also highlight the potential value of a diagnostic‐to‐intervention model [90, 91]. Rather than investing exclusively in broad communication or stress‐management programmes, healthcare organisations may benefit from first assessing unit‐specific exposure patterns, interactional demands and relevant psychological resources. This preliminary assessment can help tailor the content and allocation of preventive initiatives to units where recurrent challenging interactions coincide with greater support or capability‐development needs [83].

Brief, focused training may represent one component of this broader prevention system. Programmes may use scenario‐based practice to develop practical microskills, such as recognising escalation cues, communicating clearly, setting boundaries and applying de‐escalation techniques [4, 30, 68, 70]. These activities can provide mastery, modelling and feedback experiences relevant to interaction‐focused self‐efficacy [16].

Sustaining initial gains in self‐efficacy likely depends on organisational conditions that function as ‘resource caravan passageways’ [11]. Unit coordinators and nurse managers can contribute by providing specific and constructive feedback, modelling effective interaction strategies, facilitating access to assistance and supporting postincident reflection or debriefing [4, 16, 28, 30, 39]. Finally, joint monitoring of exposure to challenging user behaviour and perceived capability to manage such encounters may help organisations distinguish between units requiring structural risk reduction, additional managerial support, targeted capability development or a combination of these measures [11]. Training should complement, rather than replace, adequate staffing, manageable workloads, clear escalation procedures, accessible reporting systems, environmental and security measures and timely postincident support [2, 4, 45].

## 5. Conclusion

The three self‐efficacy beliefs showed different prospective patterns in relation to user incivility and occupational outcomes, consistent with the view that their relevance depends partly on the correspondence between the capability assessed and the demands under consideration. Across the separately estimated models in Study 1, self‐efficacy in managing challenging user behaviour showed the most consistent predictive pattern, with higher baseline levels associated with more favourable baseline‐adjusted relative change in perceived user incivility, psychosomatic symptoms and job satisfaction. In Study 2, this resource showed an immediate within‐group increase following brief, unit‐tailored training, whereas the secondary communication outcomes showed no statistically significant immediate change. Given the uncontrolled pre–post design, immediate postintervention assessment and modest sample, this finding provides preliminary evidence of short‐term malleability rather than causal intervention effectiveness or durable change. By using prospective diagnostic evidence to inform the selection of an intervention target, the two studies illustrate a context‐sensitive diagnostic‐to‐intervention logic. Self‐efficacy in managing challenging user behaviour represents a promising resource to assess and support as one component of broader organisational strategies for preventing user mistreatment and supporting healthcare workers’ occupational well‐being.

## Author Contributions

Conceptualisation, Elena Cavallari, Ilaria Setti and Valentina Sommovigo; methodology, Valentina Sommovigo; validation, Valentina Sommovigo; formal analysis, Valentina Sommovigo; investigation, Elena Cavallari and Valentina Sommovigo; resources, Ilaria Setti; data curation, Elena Cavallari and Valentina Sommovigo; writing–original draft preparation, Elena Cavallari and Valentina Sommovigo; writing–review and editing, Elena Cavallari, Deirdre E. O’Shea, Ilaria Setti and Valentina Sommovigo; visualisation, Elena Cavallari, Deirdre E. O’Shea and Valentina Sommovigo; supervision, Ilaria Setti and Deirdre E. O’Shea; project administration, Ilaria Setti; funding acquisition, Ilaria Setti.

## Funding

The authors declare that no funds, grants or other support were received during the preparation of this manuscript.

Open access publishing facilitated by Universita di Pavia, as part of the Wiley ‐ CRUI‐CARE agreement.

## Disclosure

All authors have read and agreed to the published version of the manuscript. All participants provided their informed consent to participate in this study and provided their consent for their anonymised data to be included in this publication.

## Ethics Statement

The study was approved by the Institutional Ethics Committee of the University of Pavia (protocol code 175/24) on 10 June 2024. The research adhered to the principles of the Declaration of Helsinki and complied with European data protection standards as outlined in the General Data Protection Regulation (GDPR). Additionally, it followed the relevant provisions of Italian privacy law, specifically Law Decree DL 196/2003 and Article 89 of EU Regulation 2016/679.

## Conflicts of Interest

The authors declare no conflicts of interest.

## Supporting Information

Additional supporting information can be found online in the Supporting Information section.

## Supporting information


**Supporting Information 1** Supporting Table 1. STROBE Reporting Checklist for Study 1.

**Supporting Information 2** TREND Statement Checklist for Study 2.

**Supporting Information 3** Supporting Table 3. TIDieR Checklist for Study 2.

## Data Availability

The data that support the findings of this study are available from the corresponding author upon reasonable request.
